# Age-based health and economic burden of skin and soft tissue infections in the United States, 2000 and 2012

**DOI:** 10.1371/journal.pone.0206893

**Published:** 2018-11-01

**Authors:** Khine Tun, James F. Shurko, Laurajo Ryan, Grace C. Lee

**Affiliations:** 1 The University of Texas at Austin, College of Pharmacy, Austin, TX, United States of America; 2 Pharmacotherapy Education and Research Center, UT Health San Antonio, San Antonio, TX, United States of America; University of South Florida, UNITED STATES

## Abstract

**Objective:**

The aim of this study was to compare the incidence of skin and soft tissue infections (SSTIs) across healthcare settings and analyze direct healthcare expenditures related to SSTIs in 2000 and 2012 in the United States.

**Methods:**

We performed a retrospective, cross-sectional analysis of nationally representative data from the Medical Expenditure Panel Surveys. Population-based incidence rates were examined for all healthcare settings that include inpatient visits, emergency department visits and ambulatory visits for SSTIs. The direct costs of healthcare services utilization were reported. Population-based prescribing rates for each antimicrobial class during ambulatory visits were compared.

**Results:**

A total of 2.4 million patients experienced an SSTI in 2000 compared to 3.3 million in 2012 (40% increase). From 2000 to 2012, the incidence of patients with at least one hospital visit for SSTIs increased 22%, ambulatory care visits increased 30%, and emergency department visits increased 40%. The incidence of SSTIs in children and adolescents declined 50% (from 150 to 76 per 10,000 person; RR = 0.51, 95% CI: 0.38–0.67; p<0.001) whereas SSTIs in older adults (> 65 years of age) increased almost 2-fold (from 67 to 130 per 10,000 person; RR = 1.94, 95% CI: 1.44–2.61; p<0.001). The annual incidence of SSTI in adults did not change significantly from 2000 to 2012 (from 84 to 81 per 10,000 person; RR = 0.96, 95% CI: 0.71–1.31; p = 0.41). The total estimated direct healthcare costs of SSTIs increased 3-fold from $4.8 billion in 2000 to $15.0 billion in 2012, largely driven by an 8-fold increase in ambulatory expenditures for SSTIs. Total population-based antimicrobial prescription rates for SSTIs increased 4-fold from 2000 to 2012 (from 59.5 to 250.4 per 10,000 person).

**Conclusions:**

The highest healthcare utilization for SSTI treatment occurred in the ambulatory care setting and also accounted for the largest increase in overall direct expenditures from 2000 to 2012.

## Introduction

In the United States, skin and soft tissue infections (SSTIs) are some of the most common infectious diseases encountered across healthcare settings [1]. Published reports have suggested that the incidence of SSTIs has increased substantially over the past two decades. From 1997 to 2005, the rate of visits to physician offices, hospital outpatient departments, and emergency departments (ED) nearly doubled, accounting for 14.2 million visits for SSTIs by 2005 [2–3]. An investigation evaluating data from the Healthcare Cost and Utilization Project National Inpatient Sample (HCUP) identified a 30% increase in hospital admissions due to SSTIs from 2000 to 2004. HCUP data also identified SSTIs as the most rapidly increasing indication for hospitalizations between 1997 and 2007 [4]. A study of ambulatory and inpatient data found that the steady increase in SSTI incidence was sustained from 2005–2010 in persons younger than 65 years [5]. Recent studies evaluating the trends of SSTIs across all healthcare settings and age-groups are limited.

The costs associated with the management of SSTIs can be substantial [3–8]. In 2008, Marton et al. reported that the overall mean cost of a *Staphylococcus aureus* SSTI episode among inpatients and outpatients was $8,865 [6]. Itani et al. estimated that the median charge for a *S*. *aureus* associated SSTI hospitalization was $19,894 [8]. This study also identified a parallel 30% increase in hospital expenditures with the rise in SSTI visits from 2001 to 2009, implying an increase in $4 billion over the study period [3].

Hospitalizations for SSTIs comprise only a fraction of the overall burden of SSTIs, yet there are limited information of the clinical and economic burdens in other healthcare settings. There are no recent studies employing an overall approach to simultaneously investigate the rate and costs associated with SSTIs in the inpatient, ED, and ambulatory settings. Therefore, we conducted a nationwide population-based study to compare the incidence of SSTIs across healthcare settings and analyze direct healthcare expenditures related to SSTIs between the years 2000 and 2012. An additional aim was to describe changes in antimicrobial prescribing for SSTIs.

## Materials and methods

### Data source

We analyzed data from the Medical Expenditure Panel Survey (MEPS) administered by the Agency for Healthcare Research and Quality [9]. The MEPS is a national set of surveys of individuals, families, their medical providers and employers. It provides national estimates of healthcare use, expenditures, payment sources and health insurance coverage for the U.S. civilian non-institutionalized population on an annual basis. The MEPS uses an overlapping panel design, with new panel of approximately 35,000 respondents added each year. Data are collected in five rounds of interviews, and include the healthcare utilization events for a specific timeframe. These five interview panels cumulatively cover a two-year period.

The MEPS includes three main components: the House-hold Component (HC), the Medical Provider Component (MPC) and the Insurance Component. The MEPS HC contains information on families and individuals regarding demographics, health conditions, health status and healthcare expenditures. The MEPS MPC includes information provided by a sample of medical providers that supplements the information acquired from the house-hold interviews. Information in the MEPS MPC includes dates of visits, diagnosis and procedure codes, charges and payments. The Pharmacy Component, a sub-component of the MEPS HC, contains ‘Prescribed Medicines’ files. Medication information is obtained from survey respondents, and additional details are imputed from data obtained from pharmacy data (for example: National Drug Codes, medication name, date filled and payment sources). Each record in the file represents a unique prescribed medicine event and purchase by the household respondent or member. Expenditures, medical conditions and household characteristics related to prescribed medicine are also provided.

### Study design and definitions

We performed a retrospective cross-sectional analysis of SSTI incidence and directly related expenditures in 2000 and 2012 as well as outpatient antibiotics prescriptions for 2000, 2007 and 2012. During the MEPS interview, survey respondents are asked to report health conditions, and these conditions are then coded by professional coders using the Clinical Classification Software (CCS) codes. These codes are recorded and reported at the person-level in conjunction with medical events, including ambulatory visits and antibiotic purchases. Ambulatory visits include both outpatient and office-based visits. We assessed SSTI incidence at each healthcare setting that used CCS code 197 (skin and subcutaneous tissue infection). We identified characteristics of patients with SSTIs using the MEPS HC. Expenditures in MEPS were defined as payments from any payment source for hospital inpatient care, ambulatory care provided in offices or hospital outpatient facilities, care provided in ED, and the retail purchase of prescribed medications. The direct healthcare expenditures are reported as U.S dollars per 1 billion. We used the National Drug Code directory, generic names, and the Multum Lexicon therapeutic classification database (Cerner Multum, Inc) to identify and classify antimicrobials. Antimicrobials for SSTIs were defined as events of antimicrobial purchase linked to CCS code 197.

### Data and statistical analyses

SSTI incidence rates were defined as the annual number of SSTI events divided by the corresponding population estimate. Population denominators were derived using the MEPS HC. The MEPS estimate U.S. populations based on sampled persons in the target population (civilian, non-institutionalized) for the entire year. Prior to 2001, population estimates were based on 1990 census data, but in 2001, MEPS transitioned to 2000 census-based population estimates for post-stratification and ranking;. The sample design of the survey includes stratification, clustering, multiple stages of selection and disproportionate sampling. The MEPS also use sampling weights to reflect adjustments for survey nonresponse and adjustments to population control totals from the Current Population Survey [9]. The U.S. civilian non-institutionalized population estimates derived from MEPS was 275,158,755 in 2000 and 309,875,841 in 2012. These numbers were used to calculate SSTI incidence rates per 10,000 person at each healthcare setting. Risk ratios (RR) and 95% confidence intervals (CI) were calculated using Chi-square tests to compare SSTI visits rates between 2000 and 2012.

Expenditure data for 2000 were adjusted to 2012 dollars using the Consumer Price Index. These data were reported per billion dollars. We conducted analysis comparing the expenditures for SSTIs by healthcare service reported between 2000 and 2012. Generalized linear models were used to estimate adjusted mean expenditures for each healthcare setting. We further performed similar analysis of average healthcare expenditure for SSTIs across three age groups: children and adolescents (< 18 years), adults (18 to 64 years), and older adults (≥ 65 years).

SSTI visit-based prescribing was determined as described in study design and definition. Antimicrobial prescription data were limited to antimicrobial events that occurred during an SSTI ambulatory visit. ‘Major antibiotic groups’ included penicillins, cephalosporins, clindamycin, tetracyclines, trimethoprim-sulfamethoxazole, and fluoroquinolones. Antibiotics with activity against methicillin-resistant *S*. *aureus* (MRSA) or ‘Anti-MRSA antibiotics’ included clindamycin, tetracyclines, and trimethoprim-sulfamethoxazole. We excluded any antibiotic that was reported as a topical route of administration. SSTI outpatient prescription rates were defined as the annual number of ambulatory-visit based antimicrobial prescriptions for SSTIs divided by the overall U.S. civilian non-institutionalized population estimates mentioned above. SSTI ambulatory-visit based prescription rates for each antimicrobial class in the first study year (2000) reported per 10,000 years were compared with the second study year (2007) and the final study year (2012). Risk ratios and 95% CIs were also calculated using Chi-squared tests to compare antimicrobial utilization across study years.

To account for MEPS’s complex study design, all analyses were adjusted using weights, clustering and stratification. SPSS 22.0 (IBM Crop, Armonk, NY, USA) was used for all statistical analyses. Statistical significance was determined using a two-tailed p-value of < 0.001 and 95% CI.

## Results

### Baseline characteristics of persons with SSTIs

A total of 2.4 million patients experienced an SSTI in 2000 compared to 3.3 million in 2012. Table 1 describes the characteristics of persons with SSTIs in 2000 and 2012. SSTIs in children and adolescents declined 50% (from 150 to 76 per 10,000 person; RR = 0.51, 95% CI: 0.38–0.67; p<0.001) whereas SSTIs in older adults (≥ 65 years of age) increased almost 2-fold (from 67 to 130 per 10,000 person; RR = 1.94, 95% CI: 1.44–2.61; p<0.001). The annual incidence of SSTI in adults did not change significantly from 2000 to 2012 (from 84 to 81 per 10,000 person; RR = 0.96, 95% CI: 0.71–1.31; p<0.41).

**Table 1:** Characteristics of persons with SSTIs in the U.S., 2000 and 2012.

Characteristics	2000	2012
**Persons with ≥ 1 visit for SSTI**	2,372,299	3,297,241
**Age (SD)**	41 (25)	42 (24)
**Male sex, n (%)**	1,113,312 (45.2%)	1,286,122 (47.3%)
**White race, n (%)**	2,155,667 (88.1%)	2,303,056 (84.7%)
**Hispanic, n (%)**	232,694 (9.5%)	369,794 (13.6%)
**Region, n (%)**		
Northeast ^a^	566,604 (23.2%)	546,438 (20.1%)
Midwest ^a^	682,491 (27.9%)	693,165 (25.5%)
South ^a^	685,560 (28.0%)	1,005,607 (37.0%)
West ^a^	479,528 (19.6%)	473,865 (17.4%)

^a^ P-value < 0.0001 for all the comparisons between 2000 and 2012.

### SSTI visits

**Fig 1:**
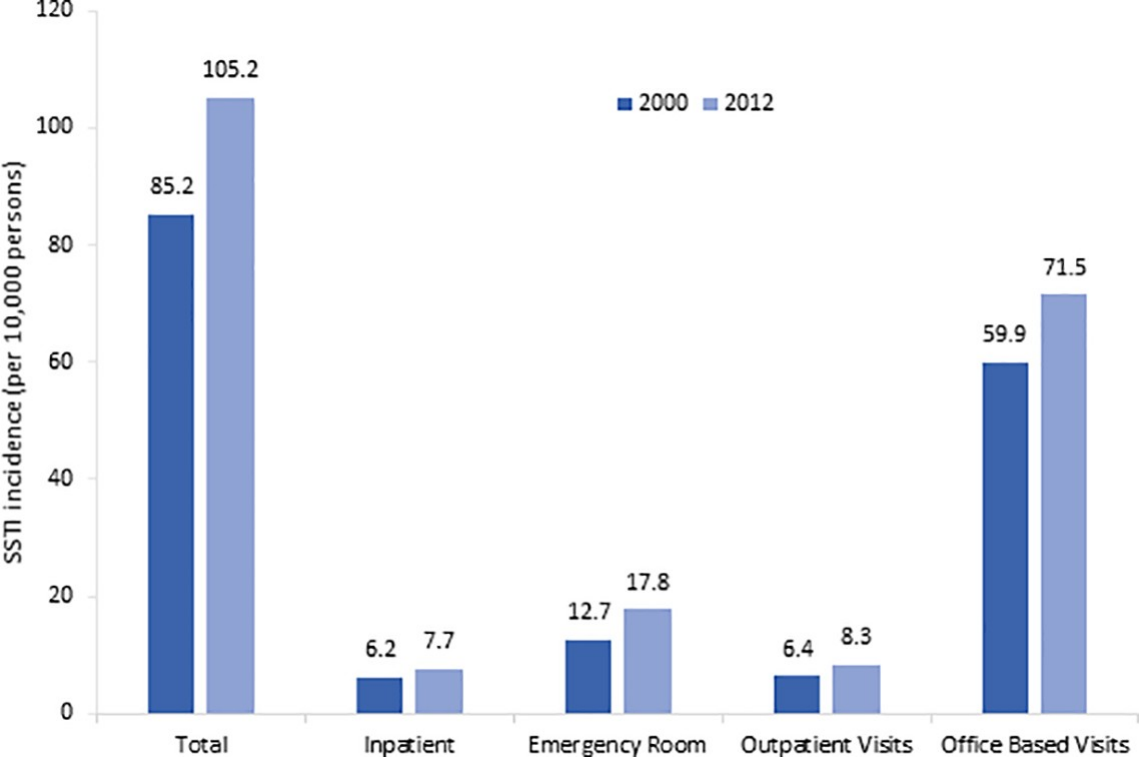
Incidence of SSTIs by healthcare setting, 2000 and 2012.

From 2000 to 2012, the national SSTI visit rate increased 24% (from 85.2 to 105.2 per 10,000 person) (Fig 1). The increase was seen across all healthcare settings: inpatient 22% (6.2 to 7.7), ED 40% (12.7 to 17.8), outpatient 30% (6.4 to 8.3), and office based visits 20% (59 to 72). In 2012, ambulatory based SSTI visits accounted for the majority of all SSTI visits (76%), followed by ED visits (17%), and inpatient visits (7%).

### SSTI direct expenditures

**Fig 2:**
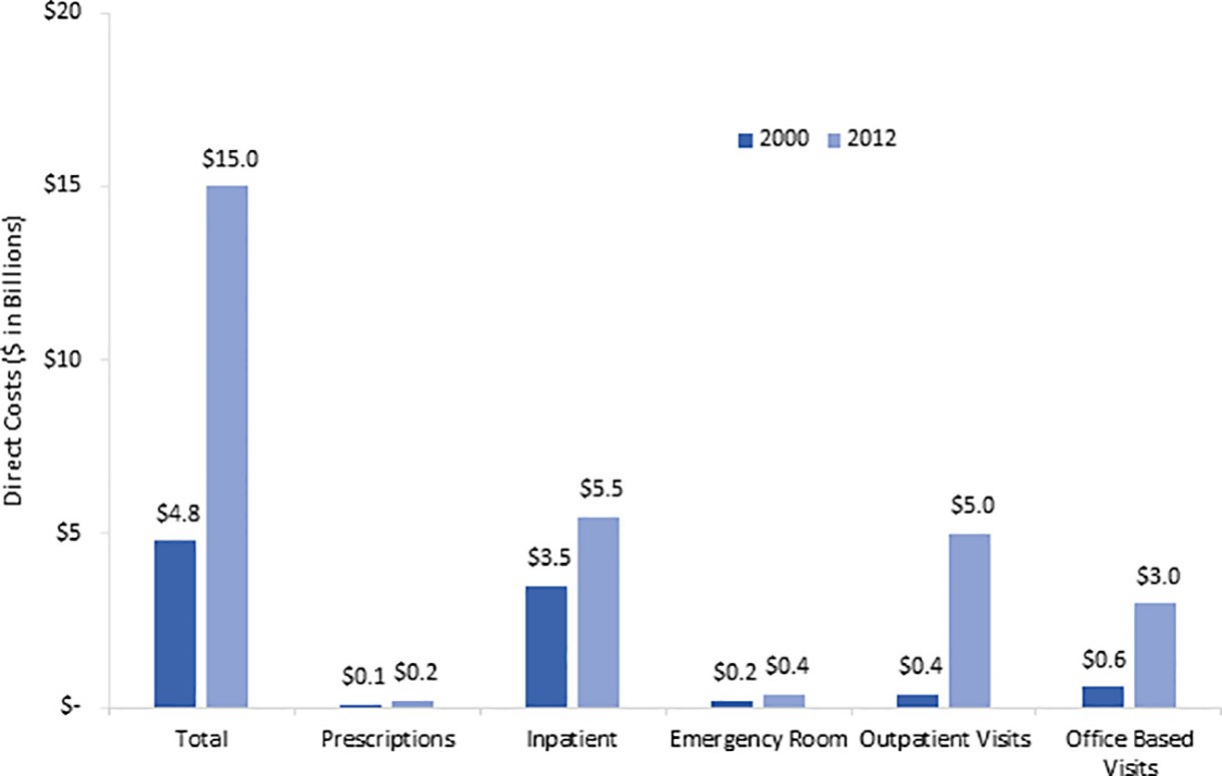
Total U.S. direct expenditures for SSTIs by healthcare service, 2000 and 2012.

The total direct expenditures for SSTI visits more than tripled from $4.8 billion in 2000 to $15.0 billion in 2012 (Fig 2). This was largely driven by expenditures in the ambulatory care setting. There was a 12-fold rise in costs associated with hospital outpatient visits, increasing from $400 million in 2000 to $5 billion in 2012. During the same timeframe, spending related to office based visits increased 5-fold from $600 million in 2000 to $3 billion in 2012. Similarly, the direct healthcare costs of ED visits doubled ($200 million in 2000 to $400 million in 2012) and that of inpatient visits increased 1.6-fold ($3.5 billion to $5.5 billion). The expenditures associated with retail prescription purchases for SSTIs doubled from $100 million in 2000 to $200 million in 2012. Inpatient visits accounted for the largest share of costs, comprising approximately 33% of total direct healthcare costs associated with SSTIs in 2012.

The average costs per ambulatory visit increased 4-fold from $253 [standard error (SE) ± $33] to $1,336 (SE ± $240) (p<0.001). The average cost for hospitalizations due to SSTI increased slightly from $20,135 (SE ± $3,997) to $22,706 (SE ± $5,234) per person. When the overall average expenditures between 2000 and 2012 were analyzed by age groups, significant increases were observed across all age groups: children and adolescents ($1,480 vs $3,292), adults ($3,302 vs $5,889), and older adults ($471 vs $1,104).

### SSTI antimicrobial utilization

**Fig 3:**
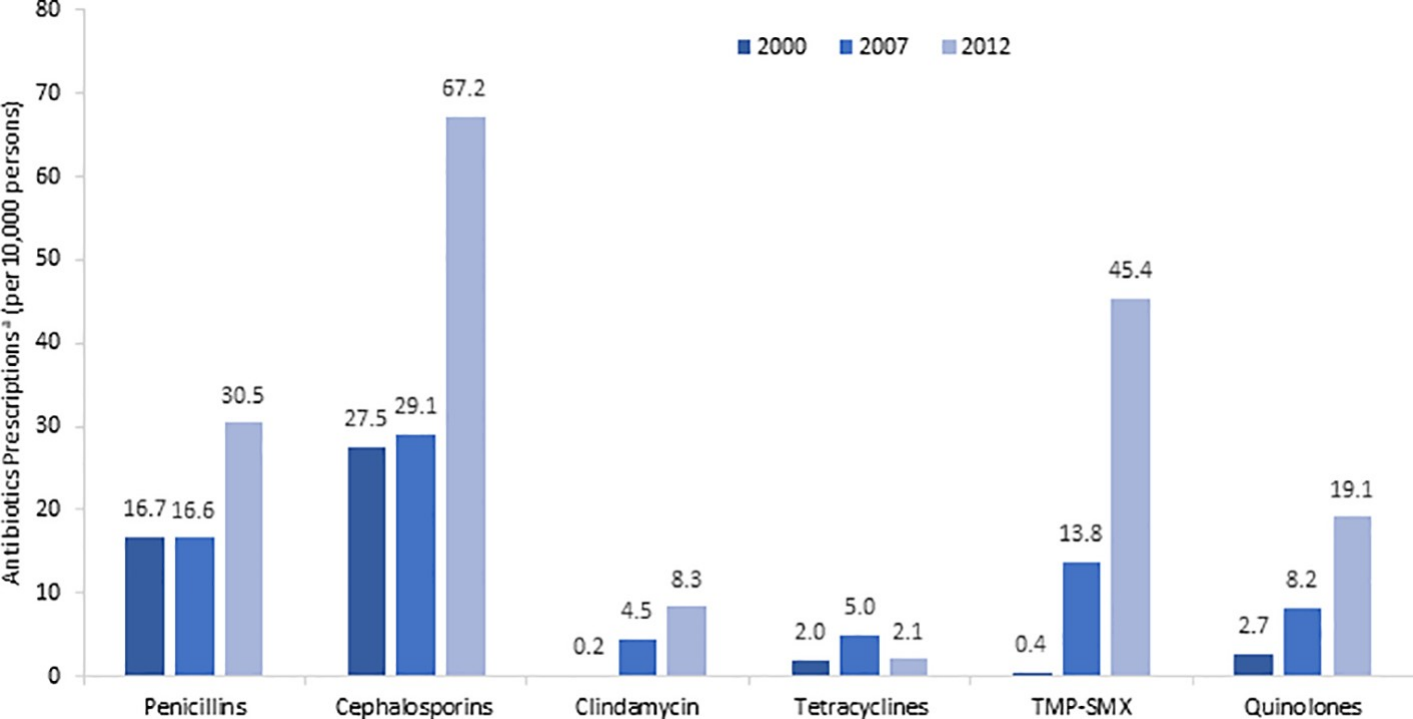
Most frequently prescribed antibiotics for SSTIs, 2000, 2007 and 2012. TMP-SMX = sulfamethoxazole-trimethoprim.

The annual number of antimicrobial prescriptions for SSTIs more than doubled from 1.6 million in 2000 to 3.3 million in 2012. Total population-based antimicrobial prescription rates for SSTIs increased 1.8-fold from 2000 to 2007 (from 59.5 to 105.9 per 10,000 person), and quadrupled from 2000 to 2012 (from 59.5 to 250.4 per 10,000 person). Fig 3. describes prescription rates for the most commonly prescribed antibiotics for SSTIs in 2000, 2007 and 2012. Cephalosporins were the most prescribed antimicrobial for SSTIs and continued to increase across the study years (27.5 to 29.5 to 67.2 per 10,000 persons). Prescriptions for antibiotics targeting MRSA substantially increased including sulfamethoxazole-trimethoprim (31-fold, 104-fold) and clindamycin (19-fold, 36-fold).

## Discussion

This study described the incidence and direct expenditures for SSTIs in the U.S. During 2000 and 2012, visits for SSTIs, as well as total direct healthcare costs, increased substantially across healthcare settings.

There was a notable 24% increase in the overall incidence of SSTI from 2000 to 2012. This trend is consistent with a prior report by Hersh *et al*. that demonstrated a 50% increase in SSTI incidence using the National Ambulatory Medical Care Survey and the National Hospital Ambulatory Medical Care Survey from 1997 to 2005 [2]. A subsequent study by Miller *et al*. reported that SSTI visit rates in adults under 65 years remained constant from 2005 to 2010 in both ambulatory and inpatient settings [5]. Our findings demonstrated that there were opposing trends in different age groups. While the incidence of SSTI visits decreased in children and younger adults, there was no change among adults, and older adults (≥ 65 years) experienced an doubling of rates. Recent U.S. census reports showed not only are the number of older adults growing, but are living longer than ever before. Changes to aging skin and deteriorating immune status to resist infections increases susceptibility of SSTIs in older adults. Furthermore, higher rates of nursing home and hospital visits among older adults increases risk of colonization with drug-resistant pathogens such as MRSA that may predispose to subsequent SSTIs. Together, with an increasingly aging population, these factors might have largely contributed to the increasing rates of SSTIs in this group.

Consistent with prior studies, this study identified the relatively high volume of SSTIs managed in the ambulatory care setting [10–13]. In 2012, the majority (76%) of all visits for SSTIs occurred in the ambulatory care setting, accounting for 2.5 million visits annually, a 30% increase since 2000. Miller et al. estimated 95% of SSTIs among patients less than 65 years were diagnosed in in the ambulatory setting in 2010 [5]. Prior studies have described the impact of the emergence and spread of community-associated MRSA (CA-MRSA) strains as a major cause of skin infections in the ambulatory setting since 2000 [11–15]. While the present study did not evaluate microbiological data, the overlap of the rise of CA-MRSA over the study period might have contributed to an increase in visits in the ambulatory setting found in this study. Moreover, high recurrence rates of SSTIs also account for a large proportion of ambulatory visits for SSTIs [13,15–18]. Studies in a Texas primary care setting identified that 35% of patients with SSTIs caused by CA-MRSA reported treatment failures and 78% experienced recurrent SSTIs [15,16].

The overall healthcare costs associated with SSTIs tripled between 2000 and 2012, accounting for an economic burden of $15 billion in 2012. Our findings also indicate that while there was an increase in direct expenditures across all healthcare settings, that there was a substantial increase in the ambulatory setting, amounting to more than half of all expenditures ($8 billion). The average costs per ambulatory care visit increased 4-fold to $1,336 in 2012. This rising increase is consistent with prior studies across all age ranges [5–7]. However, the mean cost for an SSTI inpatient episode [$22,706] with previous literature with ranges from $8,865 to $40,046 [3–5], indicating that hospitalization costs have remained relatively consistent. However, further studies to determine implications in shifts in health care utilization for SSTIs are warranted. A comparative analysis of direct non-drug medical costs to manage SSTIs in hospitals versus outpatient settings by Ektare *et al*. revealed that reducing hospitalizations and shifting SSTI care to outpatient clinics could result in cost savings of more than 53% [19]. Recent studies have described outpatient parental infusion therapy as a cost-effective alternative option to hospitalizations for managing SSTIs [20,21]. These economic models assessed cost implications of transitioning SSTI care to the ambulatory care setting and predicted the impact to reduce economic burden of SSTIs.

There was an overall increase in the annual number of outpatient antimicrobial prescriptions from 2000 to 2012. The greatest increase in prescription rates were for TMP-SMX followed by clindamycin, quinolones, and cephalosporins. Over the last two decades, there has been a dramatic change in the epidemiology of SSTIs with the rise of community-associated MRSA. Studies have shown that approximately 25 to 75% of *S*. *aureus* strains associated with SSTIs were methicillin resistant [1,14]. In 2007, CDC released an algorithm for management of staphylococcal SSTIs and recommendations for empiric outpatient antimicrobial treatment plan [22]. Four years later, the Infectious Diseases Society of America published its first guideline for managing MRSA infections [23]. Adoption of these recommendations and the recognition of this changing epidemiology by ambulatory providers is reflected by our finding of increased prescribing of anti-MRSA antibiotics prescriptions (e.g. TMP-SMX, clindamycin) across three study years (2000, 2007, and 2012). Furthermore, previous epidemiological studies have associated increased fluoroquinolone use with the rise of CA-MRSA incidence rates [24]. Therefore, the rising use of fluoroquinolones for SSTIs and overall may be particularly concerning in promoting the clonal expansion of CA-MRSA strains.

There are several limitations to this study. First, we used CCS codes to identify SSTI occurrences. Relying on coding for SSTI rates is inherently subject to bias introduced by potential misclassification and coding errors. While SSTIs are frequently managed by nurse practitioners, physicians assistants, and other non-physician clinicians, this sample did not include non-physician clinician office based visits which might have underestimated the true burden of SSTIs in the ambulatory setting. This was a cross-sectional study design and might be susceptible to ecological fallacy bias and cannot assume consistent trends over the time period. The MEPS database does not provide patient related medical information, classification regarding type or severity of SSTI, or microbial etiology. Therefore, assessment of appropriate antimicrobial prescriptions is not possible. Another limitation is the source of outpatient antimicrobial prescribing data. Since the numbers are based on purchases rather than number of prescriptions that were written, the actual number of prescriptions is likely to be underestimated. Lastly, the MEPS does not include federal institutions such as federal, military or Veterans Affairs healthcare facilities, therefore our findings may not be reflective of those populations. A strength of this study is that this is one of the largest studies conducted describing a holistic perspective of the clinical and economic burden of SSTIs across all treatment settings in the U.S.

## Conclusions

In conclusion, the clinical and economic burden of SSTIs has substantially increased from 2000 to 2012. The highest healthcare utilization for SSTI treatment occurred in the ambulatory care setting and also accounted for the largest increase in overall direct expenditures from 2000 to 2012. Anti-MRSA antimicrobial prescriptions increased in the ambulatory care setting, the largest increase was for prescriptions of TMP-SMX and clindamycin.
